# Medical Communications, with the Proceedings of the Seventy-first Annual Convention of the Connecticut Medical Society, Held at Rockville, May 27th and 28th, 1863

**Published:** 1863-08

**Authors:** 


Medical Communications, with the Proceedings of the Seventy-first Annual Convention of the Connecticut Medical Society, held at Rockville, May 27th and 28th, 1863.

The present pamphlet completes volume I. of the new series, and contains the following brief but interesting papers:—

The Dignity and Grandeur of the Medical Profession; by Josiah G. Beckwith, M.D. Logic applied to Medical Science; by James C. Jackson, M.D. Vindication of Army Surgeons; by Ashbel Woodward, M.D. Calomel in Scarlatina; by Ebenezer K. Hunt, M.D. Physiology of the Chrystalline Lens, or Adjustment of the Eye to Distinct Vision at Different Distances; by Moses C. White, M.D. Sanitary Report of Hartford County; by Lucien S. Wilcox, M.D. Report of an Anomalous Surgical Case; by M. C. White, M.D. Biographical Sketch of the late Luther Ticknor, M.D.; by J. G. Beckwith, M.D. Biographical Sketch of the late Jehiel Williams, M.D.; by J. G. Beckwith, M.D.

The first article mentioned, being the Annual Address, by Dr. Beckwith, contains a condensed but very interesting resumé of medical history, and presents the high moral and scientific objects of our profession in very chaste and eloquent language.

The following, which constitutes the closing paragraphs of the address, is worthy of a perusal once a week throughout the year:—

"In contrast with this desperate progress of the age, our profession presents a noble contrast. Our progress has been steady and gradual; in the grand accumulation of its literature, in the higher standard of its attainments, it cautiously advances through long and intelligent processes of transition.

"Human life, although protected by human and divine laws, can only be committed with safety into the hands of a profession composed of men of high intelligence, of extensive learning. We observe in the history we have given of ancient medicine that one important fact stande forth prominently,—that all the great lights in the profession were men nurtured in the schools and educated in the colleges and universities of the day. Hence the researches and discoveries made by them, of which we receive the benefit. With learning, must be combined strong common sense, a retentive memory, and sound discriminating judgment. He must be impressed that he has an important work to accomplish, requiring intense labor, study, and observation to make it useful to the world. He must be a man of large brains and broad sympathies,—broad enough to embrace the whole human family. The body must be educated as well as the mind; he should be strong for toil, and capable of enduring the inspiration of the mind. Such men are not usually fanatical, but useful and practical. They do not originate narrow systems and dreamy speculations, but substantial improvements and real reforms, based upon scientific research. The physician of this age must be eminently practical as well as liberal in his views. He must be a sort of balance-wheel to regulate the social system. He must be a patient man in the best sense of the word, a *gentleman*, kind, courteous, obliging, modest, generous, and genial; conceding, forbearing, holding fast and loving all things good; not stubborn, but maintaining a manly independence. Such a man possesses the elements of moral greatness, and will exert a healthy influence over these stormy and perilous times. He will be useful to the profession and to the world, inspiring confidence and nurturing hope, evolving light out of darkness and dispelling the gloom which prevades the chamber of death with the celestial rays which radiate from the great centre of light and happiness. His faith will be strong from intelligent research in a system of medical practice which has a literature and history of which the world may be justly proud, being the observations of more than three thousand years, reviewed, corrected, and tested by the experience of men of the greatest learning in the profession, men of profound research, and the discoveries in science and the arts during this whole period of time, of all the scientific men who belong to our brotherhood. And the march of improvement must still continue to be upward and onward. Constant contributions are being made to its literary wealth from the scientific researches of its hundred colleges and universities on both the continents, and by the observations of the thousands engaged in the practical duties of the profession. Higher standards of excellence and greater perfection in all the departments of medicine will yet be reached. These considerations will encourage every member of this venerable Society to do his whole duty in that noble cause to which he has dedicated himself during his brief day of labor; and in the consummation of this material world.

'"When the cloud-capped towers, the gorgeous palaces,
Nay, the great globe itself shall be dissolved,
And like the baseless fabric of a vision,
Leave not a wreck behind."'

We shall survive this wreck of matter and this crash of worlds. But the labors of our profession will have terminated with the annihilation of disease and death, and man's restoration to Paradise. The profession will then rest from their labors and enter upon the reward of enduring faithfulness to suffering humanity."

